# Recovery of Copper and Zinc from Livestock Bio-Sludge with An Environmentally Friendly Organic Acid Extraction

**DOI:** 10.3390/ani14020342

**Published:** 2024-01-22

**Authors:** Kuang-Wei Yen, Wei-Chen Chen, Jung-Jeng Su

**Affiliations:** 1 Department of Animal Science and Technology, National Taiwan University, Taipei 10673, Taiwan; 2 Bioenergy Research Center, College of Bio-Resources and Agriculture, National Taiwan University, Taipei 10617, Taiwan

**Keywords:** livestock sludge, acetic acid extraction, zinc, copper, sustainable assay, heavy metals, residue, bioaccumulation, Taiwan

## Abstract

**Simple Summary:**

In Taiwan, high concentrations of Cu- or Zn-rich livestock sludge can result in high Cu/Zn residues in compost intended for land applications. This study aims to recover Cu and Zn from the sludge after the livestock wastewater treatment process through extraction with organic acids or H_2_O_2_/organic acids. The results showed that the best removal efficiency for Cu (40%) and Zn (70%) from the concentrated livestock bio-sludge was achieved using certain concentrations of acetic acid in a 48-h reaction time. This study demonstrates an eco-friendly method for extracting Cu and Zn, making livestock sludge more viable for land application.

**Abstract:**

Pig farmers in Taiwan tend to overdose copper (Cu) and zinc (Zn) in animal feeds to ensure pig health. The application of Cu- or Zn-rich livestock compost to fields can result in high Cu/Zn residues in surface soil and violate limitations for zinc and copper in land applications. This study aims to extract Cu and Zn from sludge using organic acid or H_2_O_2_/organic acids. The livestock bio-sludge was dried and treated with different concentrations of acetic acid (1N, 2N, and 4N). The acid-extracted sludge was then treated with or without adding H_2_O_2_ during different periods (4, 24, and 48 h) to investigate the efficiency of acid extraction of Cu and Zn. The supernatant of the acid-extracted product was separated from the residues through centrifugation. Experimental results showed that the treatment set of dried bio-sludge with 2% H_2_O_2_ significantly promoted the removal efficiency of Cu and Zn from the bio-sludge (*p* < 0.01). The best removal efficiency of Cu and Zn from the bio-sludge was 40% and 70%, respectively, using 4N acetic acid in the 48 h group. The study shows a green method for extracting Cu and Zn from livestock sludge, enhancing the sustainability of intensive livestock farming.

## 1. Introduction

The common heavy metals in domestic sewage sludge include zinc (Zn), copper (Cu), chromium (Cr), lead (Pb), nickel (Ni), and cadmium (Cd) [1,2,3,4,5]. Livestock sludge, rich in copper and zinc primarily from animal manure, also contains traces of Pb and Cr, accumulating through microbial proliferation in wastewater treatment [6,7]. This nutrient-rich sludge is commonly used as fertilizer. Cu and Zn are prevalent in swine feces due to their use in feed for anemia prevention and weight gain [8,9,10,11,12]. However, elevated heavy metals in fertilizers may lead to bioaccumulation in food [13]. Cu and Zn concentrations in compost from livestock waste exceed legal limits in Taiwan [14]. Land application of swine manure as fertilizer may lead to Cu and Zn buildup in the soil, affecting soil and groundwater quality and potentially impacting the food chain. Excessive Cu and Zn accumulation can hinder crop growth and increase reactive oxygen species in the human body [15,16,17]. There is a growing need for an economically viable and environmentally friendly process to remove heavy metals from livestock sludge.

The acid extraction method is commonly employed in sewage sludge treatment, utilizing acids such as nitric acid, hydrochloric acid, sulfuric acid, or organic acids like acetic acid, citric acid, and oxalic acid [18]. The acid treatment involves proton transfer to the sludge, displacing heavy metals, which then dissolve in the solution [16]. Factors influencing extraction efficiency include sludge composition, extraction time, pH, acid types, liquid-to-solid ratio, temperature, and additives [1,2,3,5,19,20,21,22,23]. Inorganic strong acids may damage the original sludge structure. Organic acids, such as acetic and citric acids, are shown to extract copper and cadmium effectively [24]. Studies demonstrate that organic acids from agricultural waste fermentation, like citric acid from *Aspergillus niger*, can remove heavy metals from sludge [25]. Research from Lithuania indicates a high removal efficiency of Cu and Zn using 0.5 M citric acid, while acetic and oxalic acids show no significant difference. However, organic acids require longer extraction times and higher concentrations compared to inorganic acids [1]. Additionally, various concentrations of citric acid were used to extract Cu and Zn in sewage sludge at pH = 3–4. The removal efficiency of Cu and Zn was about 60–70% and 90–100%, respectively. However, the results of organic acids still require a relatively long time and higher concentration of acids than those of inorganic acids (sulfuric acid, nitric acid, and hydrochloric acid) [20].

In summary, organic acids have a certain potential to extract heavy metals in sludge. The advantages are the higher biodegradability and mildly acidic conditions of the organic acids, which are conducive to proceeding with the biosorption of Cu and Zn by yeast. The objective of this study is to investigate the efficiency of the acetic acid extraction process in removing Cu and Zn from the bio-sludge by adjusting extraction conditions, hoping to develop an environmentally friendly protocol to extract Cu and Zn from livestock sludge.

## 2. Materials and Methods

### 2.1. Collection and Preparation of the Livestock Sludge

The sludge samples were collected from the sludge gravity concentrator of the National Taiwan University (NTU) livestock farm in Taipei City. The main source of the sludge was a piggery wastewater treatment facility that utilized anaerobic digestion and activated sludge treatment. The collected sludge samples were dehydrated in an oven at 105 °C for 8 h and then ground to achieve a particle size of 20 mesh (about 0.84 mm) for further study.

### 2.2. Extraction of Copper and Zinc from the Sludge by Adding Acetic Acid and Hydrogen Peroxide

**Figure 1:**
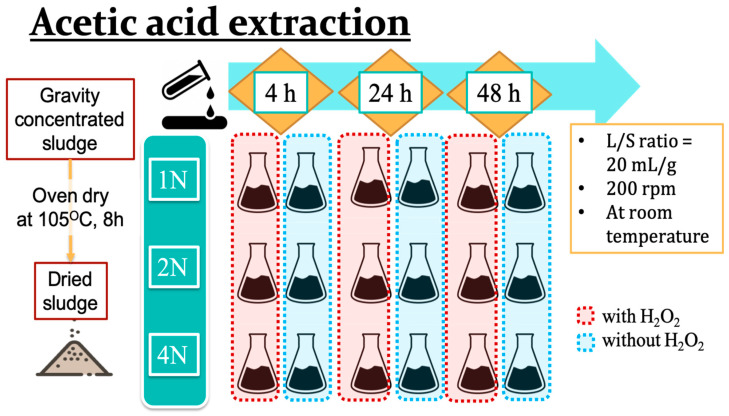
The process scheme for acetic acid extraction of heavy metals from livestock sludge.

To establish the optimal parameters for acidic copper and zinc extraction from livestock sludge samples, the focus was on the concentrations of acids, the addition of hydrogen peroxide, and the duration of the experiments. Three different concentrations of acetic acid, 1N, 2N, and 4N, were applied with or without the addition of hydrogen peroxide. The initial design for the acidic extraction using the addition ratio was sludge powder:acetic acid:hydrogen peroxide = 1:20:2, and the duration times were 4, 24, or 48 h with steady, stirring at 200 rpm under ambient conditions (Figure 1).

The extraction experiment was designed by referring to Zaleckas’s study [1]. Dried sludge powder (7.5 g) was mixed with 150 mL of acetic acid (H_3_COOH, Fisher Scientific, Leicestershire, UK) in a 250 mL beaker, and then 15 mL of hydrogen peroxide (2% H_2_O_2_, ECHO Chemical Co., Ltd., Miaoli, Taiwan) was added. The mixture with a paraffin cover was then placed on a hot plate stirring at 200 rpm for an acidic reaction under ambient conditions. Once the reaction was complete, the liquid mixture was then transferred into 50 mL centrifuge tubes and centrifuged at 3000 rpm for 15 min. After centrifugation was complete, the supernatant was transferred to clean 50 mL capped tubes for further study, and the residues were rinsed with deionized water and re-centrifuged at 3000 rpm for 15 min three times to eliminate acetic acid solution. The final residue was then dehydrated in an oven at 105 °C for 8 h and ground to achieve a particle size of 20 mesh for the quantitative analysis of copper and zinc using Flame Atomic Absorption Spectroscopy.

### 2.3. Quantitative Analysis of Heavy Metals

Crucibles were rinsed with deionized water and then heated at 105 °C and put into the ashing furnace at 600 °C for 2 to 4 h. The sludge samples were ashed in triplicates by placing them into the ashing furnace at 600 °C for 6 to 8 h. The samples were mixed and heated with 5 mL of 3N HCl (Fisher Scientific, UK) until the solid samples were completely dissolved in an HCl solution. When the mixture of samples and HCl was cooled down to room temperature, the mixture was filtered with filter paper (Advantec No.1 125 mm, Toyo Roshi Kaisha, Ltd., Tokyo, Japan) and prepared in a 100 mL volumetric flask for further analysis of heavy metals using a flame atomic absorption spectrometer (AAnalyst 200, PerkinElmer, Inc., Waltham, MA, USA).

### 2.4. Analysis of Liquid Samples

Liquid samples were analyzed for chemical oxygen demand (COD) using Standard Methods [26]. Samples were filtered, and the filtrates were analyzed for anions and cations through ion chromatography (or ion-exchange chromatography) (Metrohn ion analysis; Metrohn AG, Herisau, Switzerland) [27]. The electrical conductivity (EC) of liquid samples was determined with a conductivity meter (ExStik EC500, EXTECH Instrument, FLIR Commercial Systems, Goleta, CA, USA). The pH of liquid samples was determined using a pH meter (PH200, CLEAN Instruments, Shanghai, China) after calibration with standard solutions.

### 2.5. Statistical Analysis

The statistical analysis for this trial used SAS^®^ 9.4 (SAS Institute Inc., Cary, NC, USA) and then Prism 6 (GraphPad Software Inc., San Diego, CA, USA). Statistical analysis uses a 3 × 2 × 3 factorial design experimental design, similar to our previous study [28], comparing the interrelationship and sympathetic effects of three variables (acetic acid concentration, 2% hydrogen peroxide addition, and reaction time). If the results from the variance analysis are significant, Tukey’s honest significant difference (HSD) test would be used to compare the difference among the various factor grades. The 18 groups were compared for means between treatments using the least square mean if significant differences were achieved. The figures and tables were generated based on means and standard deviations and marked a significant difference when *p* < 0.01.

## 3. Results and Discussion

### 3.1. Analysis and Extraction of Heavy Metals from Gravity-Concentrated Sludge Samples

The physicochemical properties of gravity-concentrated sludge were analyzed. The concentrations of copper and zinc were 528.1 ± 12.1 and 1347.3 ± 54.1 mg/kg, respectively, which are higher than the Taiwanese heavy metal limits for fertilizers. The rich content of phosphorus (42,468 ± 5176 mg/kg) and potassium (1914 ± 377.8 mg/kg) make the sludge a valuable fertilizer for fitting the plants’ growth requirements. However, the concentrations of lead (8.1 ± 7.7 mg/kg) and chromium (18.5 ± 1.9 mg/kg) are of great concern in environments. The moisture content of gravity-concentrated sludge is 95.8 ± 1.83% (Table 1).

### 3.2. Different Combinations of Acetic Acid with Hydrogen Peroxide for the Extraction of Copper from Sludge Vary with Reaction Periods

This experiment for copper extraction is a three-factor design with three different acetic acid concentrations (1N, 2N, and 4N), three different treating times (4, 24, and 48 h), and with and without adding hydrogen peroxide. According to the results of three-way ANOVA, all three main effects (acetic acid concentrations, extraction times, and hydrogen peroxide addition) are individually significant (*p* < 0.01), except for extraction time. The interaction between acetic acid concentrations and hydrogen peroxide addition is significant (*p* < 0.01), as well as the interaction between acetic acid concentrations and extraction time (Table 2).

**Figure 2:**
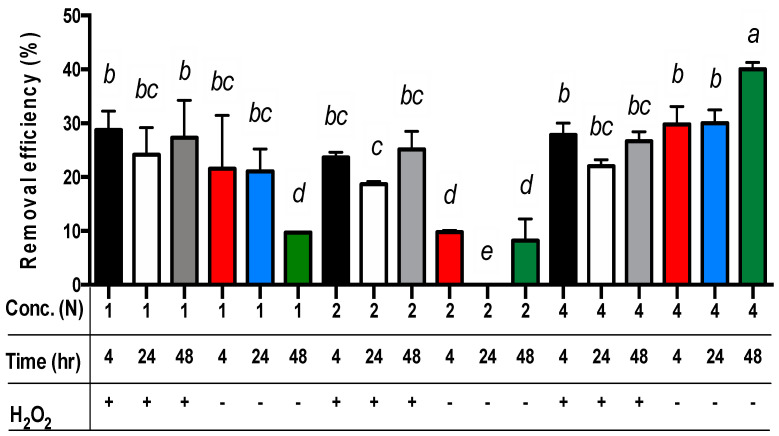
The removal efficiency of copper from livestock sludge with different concentrations of acetic acid (1N, 2N, and 4N) and with or without (+ and −) the addition of H_2_O_2_ during different periods (4, 24, and 48 h). The error bar is the standard error of the mean (*n* = 3). Different letters between the bars indicate the significant difference in the least square means at the 1% level. Percentage data were square-root transformed before analysis.

For all three main effects, the best copper extraction results from sludge samples were achieved by applying 4N acetic acid (Table 3). The copper concentrations in the residues differed significantly, measuring 372.0, 408.7, and 453.2 mg/kg for the use of 4N, 1N, and 2N acetic acid, respectively. The removal efficiency of copper from the sludge was 29.6%, 22.6%, and 13.7% when using 4N, 1N, and 2N acetic acid, respectively (*p* < 0.01). For the extraction time, the removal efficiency of copper from the sludge was 23.7%, 23.2%, and 18.8% for 4, 48, and 24 h, respectively (Table 3). Only the removal efficiency of copper from sludge in 24 h was significantly different from the sets in 4 h or 48 h (*p* < 0.01). However, the removal efficiency of copper from sludge in 4 h or 48 h was not significantly different. Moreover, the removal efficiency of copper from sludge with the addition of hydrogen peroxide (24.7%) was significantly different from the sets without hydrogen peroxide (18.8%) (*p* < 0.01) (Table 3). Among these data, the removal efficiency of copper from sludge with the addition of hydrogen peroxide in the sets of 1N or 2N acetic acid was significantly different from the sets without hydrogen peroxide (*p* < 0.01) (Table 3 and Figure 2). Nevertheless, the removal efficiency of copper from sludge with the addition of hydrogen peroxide in the set of 4N acetic acid was lower than the sets without hydrogen peroxide (Figure 2).

Surprisingly, the removal efficiency of copper from sludge without the addition of hydrogen peroxide in the set of 4N acetic acid is still the highest among all other sets (*p* < 0.01) (Figure 2). This may imply that hydrogen peroxide was limited under lower pH conditions for copper extraction (e.g., 4N acetic acid).

For the comparison of acetic acid concentrations and extraction time without the addition of hydrogen peroxide, the best removal efficiency of copper from the sludge was achieved through the sets of 4N acetic acid for 48 h (*p* < 0.01) (Figure 2). The worst removal efficiency of copper from the sludge without the addition of hydrogen peroxide was observed in the sets of 2N acetic acid for 24 h (*p* < 0.01) (Figure 2). However, in comparison of acetic acid concentrations and extraction time with the addition of hydrogen peroxide, there were no significant differences in the removal efficiency of copper from the sludge among all sets besides the sets of 2N acetic acid for 24 h extraction time (*p* < 0.01) (Figure 2).

Finally, the least square means multicomparison (α = 0.01) showed that the sets of 4N/48 h/- (i.e., 4N acetic acid concentration for 48 h extraction time without hydrogen peroxide addition) achieved the best copper removal efficiency (about 40%) (Figure 2).

### 3.3. Different Combinations of Acetic Acid with Hydrogen Peroxide for the Extraction of Zinc from Sludge Vary with Reaction Periods

This experiment for zinc extraction is a three-factor design with three different acetic acid concentrations (1N, 2N, and 4N), three different treatment times (4, 24, and 48 h), and with and without adding hydrogen peroxide. According to the results of three-way ANOVA, all three main effects (acetic acid concentrations, extraction times, and hydrogen peroxide addition) alone are significant (*p* < 0.01). The interaction between acetic acid concentrations and hydrogen peroxide addition is significant, as well as the interaction between acetic acid concentrations and extraction time (*p* < 0.01). Moreover, the interaction between hydrogen peroxide addition and extraction times is significant (*p* < 0.01) (Table 4).

**Figure 3:**
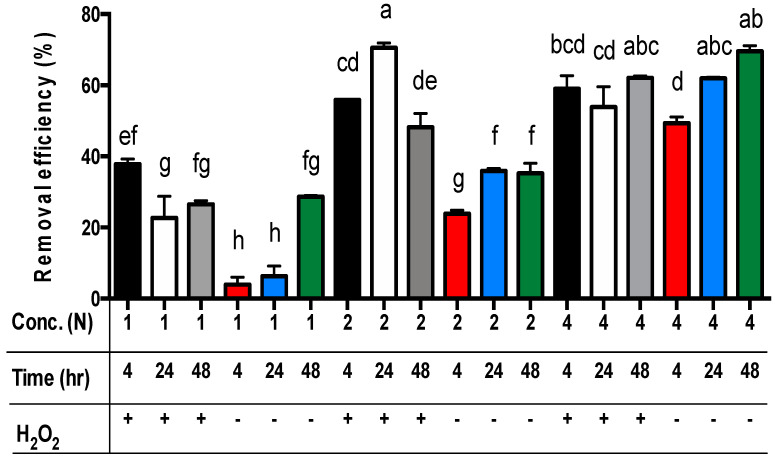
The removal efficiency of zinc from livestock sludge with different concentrations of acetic acid (1N, 2N, and 4N) and with or without the addition of H_2_O_2_ during different periods (4, 24, and 48 h). The error bar is the standard error of the mean (*n* = 3). Different letters between the bars indicate a significant difference in the least square means at the 1% level. Percentage data were square-root transformed before analysis. +: addition; **−**: no addition.

For all three main effects, the best zinc extraction results from sludge samples were achieved by applying 4N acetic acid (Table 5). The zinc concentrations in the residues were significantly different, and the removal efficiency of zinc from the sludge was 53.1%, 46.9%, and 21.3% for using 4N, 2N, and 1N acetic acid, respectively (*p* < 0.01). For the extraction time, the removal efficiency of zinc from the sludge was 46.2%, 40.6%, and 36.9% for 48, 24, and 4 h, respectively (Table 5). Only the removal efficiency of zinc from sludge in 48 h was significantly different from the sets in 4 h or 24 h (*p* < 0.01). However, the removal efficiency of zinc from sludge in 4 h and 24 h was not significantly different. Moreover, the removal efficiency of zinc from sludge with the addition of hydrogen peroxide (48.9%) was significantly different from the sets without hydrogen peroxide (36.5%) (*p* < 0.01) (Table 5). Among these data, the removal efficiency of zinc from sludge with the addition of hydrogen peroxide in the sets of 2N or 4N acetic acid was significantly different from the sets of 1N (*p* < 0.01) (Figure 3). The removal efficiency of zinc from sludge without hydrogen peroxide addition among the sets of 1N, 2N, and 4N acetic acid was significantly different (*p* < 0.01) (Figure 3). The worst removal efficiency of zinc is observed in the sets of 1N acetic acid without hydrogen peroxide addition.

For the comparison of acetic acid concentrations and extraction time without the addition of hydrogen peroxide, the best removal efficiency of zinc from the sludge was achieved using the sets of 4N acetic acid for 24 h (i.e., 4N/24 h/-) or 48 h (i.e., 4N/48 h/-) (*p* < 0.01) (Figure 3). The worst removal efficiency of zinc from the sludge without the addition of hydrogen peroxide was observed in the sets of 1N acetic acid for 4 h or 24 h (*p* < 0.01) (Figure 3). However, for the comparison of acetic acid concentrations and extraction time with the addition of hydrogen peroxide, the best zinc removal efficiency was achieved using the sets of 2N acetic acid for 24 h and 4N acetic acid for 24 h or 48 h (Figure 3). However, there was no significant difference among these sets (Figure 3). The worst removal efficiency of zinc from the sludge with the addition of hydrogen peroxide was observed in the sets of 1N acetic acid for 24 h or 48 h (Figure 3).

Finally, the least square means multicomparison (α = 0.01) showed that there was no significant difference in the removal efficiency of zinc among the sets of 2N/24 h/+ (i.e., 2N acetic acid concentration for 24 h extraction time with hydrogen peroxide addition), 4N-48 h/+ (69.5%), 4N-48 h/- (62.1%), and 4N-24 h/- (60.2%). However, the best zinc removal efficiency (70.6%) was achieved using the set of 2N/24 h/+ (Figure 3).

### 3.4. Different Combinations of Acetic Acid with Hydrogen Peroxide for the Extraction of Copper and Zinc Simultaneously from Sludge Vary with Reaction Periods

Results showed that the addition of 15 mL of hydrogen peroxide (2%, *v*/*v*) into a 150 mL sludge sample significantly increased the removal efficiency of copper and zinc compared with the sets without hydrogen peroxide addition (*p* < 0.01) (Table 3 and Table 5). The best removal efficiency of copper and zinc (with H_2_O_2_) with the least extraction time and acetic acid concentrations was achieved using the sets of 1N acetic acid for 4 h and the 2N acetic acid for 24 h, respectively (Figure 2 and Figure 3). However, the best removal efficiency of copper and zinc (without H_2_O_2_) with the least extraction time and acetic acid concentrations was achieved through the sets of 4N acetic acid for 48 h and 4N acetic acid for 24 h, respectively (Figure 2 and Figure 3). Results showed that adding hydrogen peroxide can reduce acetic acid concentration and extraction time.

The presence of hydrogen peroxide has a significant impact on the removal efficiency of copper and zinc. Hydrogen peroxide plays a role in this mainly by changing the valence of copper and zinc oxides or hydroxides in sludge and increasing the occurrence of their dissolution. The oxidation–reduction potential (ORP) is an indicator of the valence of heavy metal patterns and affects the mobility, solubility, and activity of heavy metals. The change in ORP can change the solubility and removal efficiency of pollutants or heavy metals. In some soil, oxidants may increase the metal removal efficiency. The factor is to reduce the amount of organometallic misfit or the release of metal adsorbed by organic matter to increase the removal efficiency of heavy metals [29]. In soil pollution prevention and control, ORP is one of the common measurement indicators. Finally, there is no determination in this experiment, so it can only be known that hydrogen peroxide plays a certain role in acetic acid extraction tests.

In addition, in past studies, hydrogen peroxide has been shown to oxidize suspended particles in industrial wastewater and is effective in removing heavy metals such as lead, zinc, and copper from industrial wastewater [21]. Therefore, this study found that hydrogen peroxide, an oxidant, is significantly helpful for extracting copper and zinc in sludge under the joint action of acetic acid.

To summarize the influences of the above effects, the overall best removal amount (total extraction amount of copper and zinc) is observed in the sets of 2N acetic acid for 24 h with hydrogen peroxide addition and the set of 4N acetic acid for 48 h. However, the sets of 2N acetic acid with 2% hydrogen peroxide for 24 h were the selected conditions for copper and zinc extraction by reducing the cost and malodor associated with higher acetic acid concentrations during the extraction process.

**Figure 4:**
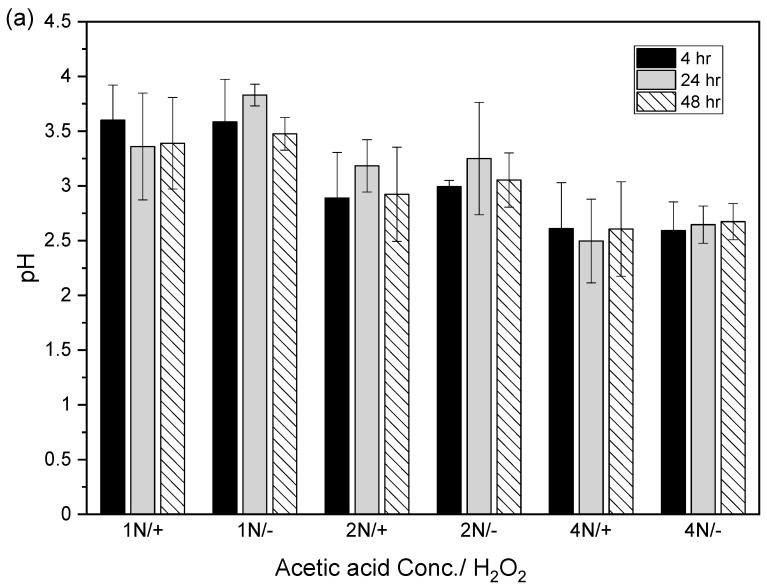

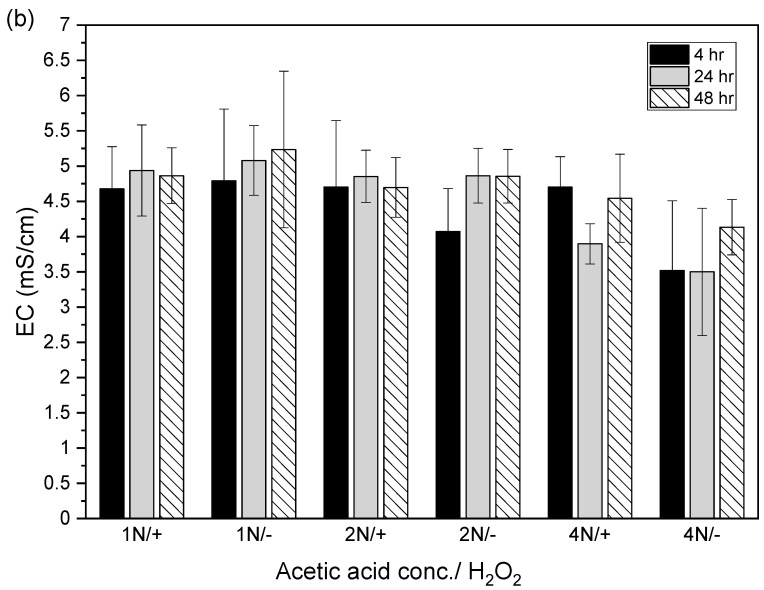
Relation between (**a**) pH value and (**b**) electrical conductivity (EC) in acid-extracted supernatant with or without 2% hydrogen peroxide solution at different acetic acid concentrations (1N, 2N, and 4N) and contact times (4, 24, and 48 h). The error bar is the standard error of the mean (*n* = 3).

After the acidic extraction of sludge samples using 1N, 2N, and 4N acetic acid, the pH values of the supernatant were 3.4–3.8, 2.9–3.3, and 2.5–2.7, respectively. The electrical conductivities using 1N, 2N, and 4N acetic acid were 4.7–5.2, 4.1–4.9, and 3.5–4.7 mS/cm, respectively (Figure 4).

### 3.5. Limitation Factors for Extracting Copper and Zinc from Sludge with A Combination of Acetic Acid and Hydrogen Peroxide

#### 3.5.1. Sources and Composition of the Sludge

The concentrations of copper and zinc in livestock sludge in this work are 528 ± 12 and 1347 ± 54 mg/kg, respectively. The zinc concentration is lower than that in sewage sludge. However, copper and zinc concentrations are higher than those in pig manure (Table 6). This disparity is due to the absorption of heavy metals from wastewater into active sludge during the wastewater treatment process [30,31]. Additionally, sludges from different sources containing other divalent metals (e.g., lead and manganese) might compete with copper and zinc for extraction solvent, leading to low removal efficiency.

#### 3.5.2. Extraction Time

The 4 h and 48 h extraction time have the best copper removal efficiency. In contrast, the best extraction time for zinc extraction is 48 h, and the second best is 24 h. The acid extraction efficiency exhibit an increased trend over time. A research team used 1N acetic acid to extract industrial waste sludge, ranging from 5 to 180 min. The results showed that the removal efficiency increased from 15% to 85% [20]. Another research team from the Netherlands chose citric acid as an extractant for sewage sludge. The analysis revealed that copper and zinc might have tight chemical fractions in the sludge, making it hard to react with citric acid. In this case, more extraction time is needed to balance the chemical reaction. The removal efficiencies of copper and zinc are 60% on day 11 and 90% on day 4, respectively. Regarding iron and calcium, they only need 1 h and 12–24 h for balancing reactions. Since both livestock sludge and sewage sludge contain large amounts of organic matter, the extraction conditions are similar. The presence of organic matter and its bonding with metals can prevent acid ions from directly binding to heavy metals in the sludge. Instead, a concentration gradient is required for proton displacement reactions with heavy metals. This process reduces chemical extraction efficiency [5]. Therefore, the lower removal efficiency of copper from the sludge in this experiment may be related to insufficient extraction time.

#### 3.5.3. Types of Acids Used

Zaleckas et al. [1] conducted a study applying different organic acids (acetic acid, oxalic acid, and citric acid) as extractants for anaerobically digested sewage sludge and trying to determine the relationships between copper, zinc, and nickel chemical fractions in the sludge and the extraction efficiencies of different organic acids with concentrations ranging from 0.01 to 1.0 mol/L. More than 50% of copper in the sludge exists in the form of organically bound fractions, while zinc is primarily bound in the form of carbonate fractions. Under conditions using 0.5 mol/L of organic acids, citric acid demonstrated the highest removal efficiency (100%), followed by acetic acid at approximately 79% and oxalic acid at only 71%. Furthermore, at concentrations ranging from 0.01 to 1.0 mol/L, increasing acid concentration and lowering pH values helped enhance zinc removal efficiency. In contrast, copper ions can form stable substances with organic matter, limiting the effectiveness of the abovementioned organic acids for removal, with citric acid being the most effective at around 50%. The author also suggests that the better removal of zinc under acidic conditions may be attributed to its predominant binding in carbonate fractions.

Wu et al. [19] applied organic acids (citric acid and acetic acid) and inorganic acids (hydrochloric acid, nitric acid, and sulfuric acid) to treat sludge from wastewater treatment facilities in printed circuit board factories. In removing copper, citric acid and acetic acid have 57% and 79% removal efficiencies, respectively. In contrast, sulfuric acid has the best removal efficiency (92%), followed by nitric acid 91%, then hydrochloric acid 81%. The performance of inorganic acids is better than organic acids. Interestingly, the copper removal efficiency of acetic acid is higher than that of citric acid.

In this study, under 2N acetic acid combined with hydrogen peroxide treated for 24 h, the copper removal efficiency is 18.7%. In contrast, the zinc removal efficiency is 70.6%, which is much better. Compared to that of citric acid, these results are poor, which might be due to the higher pH value.

#### 3.5.4. The Concentrations of Acids

The concentrations of acetic acid in this study are 1N, 2N, and 4N. The removal efficiencies of zinc were increased, followed by the rising of acetic acid concentrations. A previous study also observed similar phenomena. Acetic acid’s copper removal efficiency was 15% [1]. However, our study showed that 4N acetic acid combined with hydrogen peroxide achieved a maximum removal efficiency of up to 40%. The difference might be caused by the higher acetic acid concentration.

In another study by a Chinese and Korean research team [2], citric acid and ethylenediaminetetraacetic acid (EDTA) were used. Under the conditions of using 1 mol/L citric acid, the removal efficiency of zinc reached 90%. The highest removal efficiency for copper occurred at 0.80 mol/L citric acid, with a removal rate of approximately 49.1%, consistent with similar findings in other studies.

## 4. Conclusions

The efficiency of Cu and Zn extraction from livestock sludge is influenced by acetic acid concentration, treatment time, and hydrogen peroxide addition, with significant interactions among these factors. Optimal conditions involve using 4N acetic acid for 48 h, removing about 40% of Cu and 70% of Zn. The waste solution can be used for anaerobic digestion, generating methane and avoiding chemical pollution. The residual sludge may be considered for use as fertilizer, promoting sustainability. Future platforms may automate processing based on established parameters.

## Figures and Tables

**Table 1:** Heavy metals and physicochemical properties of gravity-concentrated sludge from NTU livestock farm.

Heavy Metals	Unit (mg/kg)
Cu	528 ± 12 *
Zn	1347 ± 54
P	42,468 ± 5176
K	1914 ± 378
Fe	5798 ± 604
Mn	493 ± 57
Pb	8.1 ± 7.7
Cd	N.D.
Cr	18.5 ± 1.9
Moisture (%)	95.8 ± 1.8
COD (mg/L)	11,357 ± 6246
pH	7.02 ± 0.13
EC (mS/cm)	1.07 ± 0.023

* Means ± S.D. (*n* = 6). N.D.: not detected. COD: Chemical Oxygen Demand. EC: Electrical Conductivity.

**Table 2:** The ANOVA table for the removal efficiency of copper from livestock sludge with different concentrations of acetic acid (1N, 2N, and 4N) and with or without the addition of H_2_O_2_ during different periods (4, 24, and 48 h) for three-factor variational analysis.

Source	Degrees of Freedom	Type III Sum of Squares	Mean Squares	F-Value	*p* Value
Acid	2	55,809.95185	27,904.97593	86.34	<0.0001
H_2_O_2_	1	12,345.88082	12,345.88082	38.20	<0.0001
Acid × H_2_O_2_	2	37,254.25877	18,627.12939	57.63	<0.0001
Time	2	5053.31842	2526.65921	7.82	0.0017
Acid × Time	4	7672.72960	1918.18240	5.94	0.0011
H_2_O_2_ × Time	2	339.12025	169.56013	0.52	0.5968
Acid × H_2_O_2_ × Time	4	6353.09868	1588.27467	4.91	0.0033

Acid: effect of acetic concentration; Time: effect of time; H_2_O_2_: effect of addition of 2% hydrogen peroxide.

**Table 3:** Comparison of the removal efficiency of copper for different variables.

Concentration (N)	Means	Difference	
4	29.6 ^a^	-	-
1	22.6 ^b^	6.95 *	-
2	13.7 ^c^	15.9 *	8.91 *
Time (h)	Means	Difference	
4	23.7 ^a^	-	-
48	23.2 ^a^	0.498	-
24	18.8 ^b^	4.89 *	4.39
w or *w*/*o* H_2_O_2_	Means	Difference	
w	24.7 ^a^	-	-
*w*/*o*	18.8 ^b^	5.97 *	-

* Significant at 1% level using Tukey’s test. Percentage data were square-root transformed before analysis. Different letters with the factors indicate the significant difference in Tukey’s test at the 1% level.

**Table 4:** The ANOVA table of the removal efficiency of zinc from livestock sludge with different concentrations of acetic acid (1N, 2N, and 4N) and with or without the addition of H_2_O_2_ during different periods (4, 24, and 48 h) for three-factor variational analysis.

Sources	Degrees of Freedom	Type III Sum of Squares	Mean Squares	F-Value	*p* Values
Acid	2	90.79026373	45.39513186	184.99	<0.0001
H_2_O_2_	1	20.76977726	20.76977726	84.64	<0.0001
Acid × H_2_O_2_	2	12.51283781	6.25641891	25.50	<0.0001
Time	2	4.17825025	2.08912513	8.51	0.0011
Acid × Time	4	8.55051418	2.13762855	8.71	<0.0001
H_2_O_2_ × Time	2	10.53066852	5.26533426	21.46	<0.0001
Acid × H_2_O_2_ × Time	4	4.340426795	1.07606699	4.39	0.0061

Percentage data were square-root transformed before analysis.

**Table 5:** Comparison of the removal efficiency of zinc for different variables.

Concentration (N)	Means	Difference	
4	53.1 ^a^	-	-
2	46.9 ^b^	6.25 *	-
1	21.3 ^c^	31.85 *	25.6 *
Time (h)	Means	Difference	
48	46.2 ^a^	-	-
24	40.6 ^b^	5.60 *	-
4	36.9 ^b^	9.34 *	3.74
W or *w*/*o* H_2_O_2_	Means	Difference	
W	48.9 ^a^	-	-
*w*/*o*	36.5 ^b^	12.37 *	-

* Significant at 1% level using Tukey’s test. Percentage data were square-root transformed before analysis. Different letters with the factors indicate the significant difference in Tukey’s test at the 1% level.

**Table 6:** Characteristics of sewage sludge (SS), pig manure (PM), pig sludge (PS), and NTU livestock sludge (LS).

Parameters	SS (*n* = 3)	SS (*n* = 4)	PM (*n* = 3)	PS (*n* = 6)	PS	LS (*n* = 6)
pH	6.10 ± 0.07	7.3 ± 0.1	8.10 ± 0.21	7.9 ± 0.1	6.42	7.02 ± 0.13
EC * (mS/cm)	1.31 ± 0.10	-	2.14 ± 0.07	-	2.91	1.07 ± 0.023
Cu (mg/kg)	347 ± 10	1735 ± 286	321 ± 13	382 ± 28	760	528 ± 12
Zn (mg/kg)	3242 ± 52	2110 ± 78	689 ± 44	3283 ± 425	4000	1347 ± 54
Pb (mg/kg)	195 ± 9.5	54.7 ± 6.2	52 ± 1.8		-	
Mn (mg/kg)				1400 ± 15		493 ± 57
Cr (mg/kg)		373 ± 24,				
References	[6]	[2]	[6]	[7]	[32]	This study

* EC: Electrical Conductivity.

## Data Availability

Data are contained within the article.
